# Exploring Women's Experiences of Information Across Their Endometrial Cancer Diagnosis and Treatment: A Qualitative Analysis

**DOI:** 10.1002/pon.70444

**Published:** 2026-04-01

**Authors:** Britta Wigginton, Abbey Diaz, Bena Brown, Joan Cunningham, Tracey DiSipio

**Affiliations:** ^1^ School of Public Health The University of Queensland Brisbane Queensland Australia; ^2^ Yardhura Walani National Centre for Aboriginal and Torres Strait Islander Wellbeing Research National Centre for Epidemiology and Population Health Australian National University Canberra Australia; ^3^ Southern Queensland Centre of Excellence in Aboriginal and Torres Strait Islander Primary Health Care Metro South Health Queensland Australia; ^4^ Menzies School of Health Research Charles Darwin University Darwin Northern Territory Australia

**Keywords:** endometrial cancer, information, information navigation, journey mapping, qualitative

## Abstract

**Background:**

Access to timely and appropriate information is a key component of quality cancer care.

**Aims:**

This study aimed to fill an important gap regarding the information experiences of women with endometrial cancer from diagnosis to treatment.

**Methods:**

This study is based on 24 interviews with women diagnosed with endometrial cancer in Queensland, Australia in 2022–2023. Reflexive thematic analysis was used to provide an in‐depth exploration of women's accounts of information across their diagnosis and treatment journeys.

**Results:**

The researchers identified three implicit and socially constructed *assumptions* women negotiated when it came to information. These included: (1) the physical body is of central importance; (2) more information is better; and (3) medical knowledge is most credible. These assumptions were not always upheld or accepted; at times, they were rejected by women and ultimately shaped how women made sense of, and spoke about, their experiences of information.

**Conclusions:**

Together, these findings point to the need for women to be supported to engage with contextually sensitive information regarding their cancer. In particular, health professionals should be actively involved in ensuring women understand their cancer (including treatment side‐effects and risk of recurrence), are supported with psycho‐social impacts, and that alternative and holistic sources of information are acknowledged alongside medical information. These findings point to the need for health professionals to reflect on their own communication practices and how they may reinforce assumptions that shape women's experiences of endometrial cancer.

## 1 Background

Uterine cancer is the most common tumor of the female reproductive organs. Endometrial cancer, which originates in the epithelial lining of the uterus, accounts for 90%–95% of uterine cancer cases [1, 2]. The incidence of uterine cancer is rising worldwide with a 15% increase in the age‐standardized rate between 1990 and 2019, likely due to the aging population and increased occurrence of risk factors such as obesity and lower parity [3]. There is no effective screening test for asymptomatic women, so detection relies on symptom recognition. While many (66%) endometrial cancer cases are diagnosed at an early stage [4], awareness of endometrial cancer and its symptoms among the general population remains low [5]. Typically, diagnosis involves multiple healthcare visits and tests. For a small proportion, diagnosis may result from presenting to an emergency department [6], which is associated with advanced stage at diagnosis and increased mortality [7]. Given the complexities of diagnosis and treatment decision‐making, women need appropriate support throughout to understand and make decisions about their cancer [8, 9].

Considering emerging literature around *patient navigation models* in cancer care [10], this article is concerned with *information navigation*. Patient information is essential in supporting cancer patients. There is evidence to suggest that meeting information needs has significant benefits to cancer patients' wellbeing including better quality of life and lower levels of depression and anxiety [11, 12, 13]. While many cancer patients prefer more information in general [11, 12, 14, 15, 16, 17, 18], needs and satisfaction vary by type of cancer, age, sex, and time since diagnosis [19, 20]. Across breast, colorectal, and gynecological cancers, studies have found that information, particularly when paired with reassurance from health professionals, can help patients regain a sense of control in the face of uncertainty, supporting emotional regulation and coping with a life‐threatening diagnosis [17, 21].

Little is known about how women diagnosed with endometrial cancer engage with and make sense of information across diagnosis and treatment. This matters given endometrial cancer is the most common gynecological cancer in high‐income countries [22], yet the physical, emotional, and informational needs of this group remain under‐researched. Evidence from the Netherlands points to lower satisfaction with cancer information among endometrial cancer survivors compared to those with lymphoma or myeloma [15, 16]. Research from New Zealand highlights erratic diagnostic pathways shaped by socio‐cultural context [23]. Gendered experiences also matter: stigma around uterine bleeding and greater trust in female doctors were noted in a recent systematic review [24]. Together, this evidence underscores the need for more direct qualitative accounts of information experiences in endometrial cancer care.

Qualitative evidence has been advocated for on the basis of directly informing the counseling of patients [8] and improving information and support during the diagnosis and treatment for endometrial cancer [24]. An emphasis on patient voice and lived experience is also reflected in cancer care guidelines [25, 26, 27, 28], which promote *person‐centered care*. Our study aimed to map the information experiences of women with endometrial cancer from diagnosis to treatment. Using a feminist framework that centers women's language and lived experiences [29], this study offers an in‐depth exploration of the taken‐for‐granted assumptions about information that women negotiated as they told their stories.

## 2 Methods

### 2.1 Study Design

This study was part of a larger, cross‐sectional mixed methods study that included a mailed self‐administered questionnaire and semi‐structured telephone interviews among women diagnosed with endometrial cancer in Queensland, Australia. This paper focuses on the qualitative findings from the interviews. Ethics approval was obtained prior to study commencement from The University of Queensland Human Research Ethics Committee (HE000046). The Consolidated criteria for reporting qualitative research (COREQ) checklist was followed [30].

### 2.2 The Research Team

Our team includes Australian researchers with content expertise in cancer epidemiology, health psychology, qualitative research, information needs in people affected by cancer, and health inequities.

### 2.3 Sample

Cancer Alliance Queensland (CAQ), which houses the Queensland Cancer Register, was used to identify potential participants. All endometrial cancer notifications received by the Queensland Cancer Register during the period 01/08/2022 to 31/08/2023 were identified. Eligible women were aged 18+ years, diagnosed with primary endometrial cancer (ICD‐10 C54.1), and living in Queensland at the time of diagnosis. Cases aged over 80 and/or living in a nursing home were excluded. All cases (*n* = 519) were identified, and a random selection (*n* = 254) was performed by CAQ staff.

Registry staff cross‐checked records for eligibility and vital status before mailing potential participants a letter of invitation, consent form and questionnaire with a reply‐paid envelope. At the end of the questionnaire, participants were invited to participate in an optional, once‐off interview to discuss their experiences in more depth. Participants who expressed interest in the interview were contacted by research staff to answer any questions and schedule an interview at a convenient time.

Of the 82 women who returned a completed questionnaire (32% response), 35 women expressed interest to participate in the qualitative interview. Two women were not contactable; one woman decided not to be interviewed when contacted; and eight expressions of interest were received following completion of interviews. In total, 24 women were interviewed. Interview participants ranged in age from 33 to 79 years (median 60 years) from across Queensland (71% major city). The median time since diagnosis was 13 months (range 5, 16) at the time of the interview.

### 2.4 Qualitative Interviews

Semi‐structured telephone interviews were conducted by BW, a female qualitative researcher with a PhD in psychology and over a decade experience in conducting qualitative health research. BW had no prior interaction with participants nor was involved in their care; participants did not know her prior to the interview. No pilot interviews were conducted. BW continued interviewing until data saturation was reached, which was defined as a richness in data that followed similar patterns and no longer provided new directions or questions [31]. We audio‐recorded interviews, which were later transcribed verbatim by a professional transcription service. No transcripts were returned to participants for feedback. Interviews lasted between 35 and 90 min (median of 65 min) and took place between November 2023 and March 2024. BW used a semi‐structured interview guide (Supporting Information S1), a patient journey map [32], and took field notes. BW found journey mapping supported notetaking throughout the interviews and helped ensure all touchpoints were addressed. Patient journey maps included the aforementioned key touchpoints, as well as how these corresponded to actions, mindset (thoughts), interactions, emotions, and reflections (Supporting Information S1).

### 2.5 Data Analysis

BW conducted data collection and analysis within a feminist methodology [29], in that interviews were participant‐directed as much as possible to afford women the space to tell their story, in their own way, on their terms—and hence analysis continued this thread of listening to, and learning from, women [33]. A feminist approach was especially important considering disclosures of medical trauma and feedback about the postal survey as restricting their capacity to freely share their experiences, with some left feeling like they were not the “right” participant we were looking for.^1^ BW took a critical, reflexive thematic analysis, studying language as a means of constructing reality, and employed both inductive and deductive approaches to analysis [34]. All analysis was handled manually (Microsoft Word) to support data management. Participants did not provide feedback on results.

Staying close to women's stories, BW took an inductive approach to understanding *what* and *how* women spoke about ‘information’.^2^ First, using the journey map structure, BW created a participant summary (using women's language direct from interviews) that mapped their information journey. This offered both a ‘birds eye view’, documenting women's cancer journey and how information featured, as well as supporting a familiarization process of the data (see Table 1). BW's analysis then further developed by engaging with her field notes, re‐listening to interview recordings and through discussions with co‐investigators (T.D. and A.D.). This led to a refined focus on *how* women shared the telling of their experiences of information. Hence, BW focused on women's language use, considering the *assumptions* implicit in how women engaged with questions and dialog around information relevant to their cancer diagnosis and treatment journeys [29, 33, 35]. Hence, BW focused on women's language use, considering the assumptions that women negotiate when telling their stories about information relevant to their cancer diagnosis and treatment journeys. Assumptions in this context are not necessarily owned by any one group (i.e., women, health professionals).

**TABLE 1:** Example of two participant summaries using the journey mapping framework as part of early data analysis.

Participant ID	Before diagnosis	Being diagnosed	Treatment	Post‐treatment	Further context	Take‐home messages
P158	Sick of bleeding all the time—went to doctor like a sixth sense all my cancers started with “there's something wrong”. I asked for a female doctor—Only reason I was diagnosed: Most male doctors say “yeah women bleed”. Learned you've got to be in charge of your own doctor‐ing.	Felt comfortable with female doctor because she found it. Had to leave job because too emotional. It's all luck I got diagnosed. As soon as they said cancer to me, my whole mind went blank.	Take daughter to all appointments—Recommends bringing someone who can hear and remember what is said. Daughter says I have a habit of forgetting stuff, only picking out what you want to hear.	Quick recovery. Started bleeding again 2 weeks ago. Back to square one—Every time book with female doctor she cancels. Don't understand what to do now—Do I go back to doctor or gynecologist?	Daughter having same issues with bleeding and she's struggling to find a doctor to listen	• Women have gotta listen to themselves and their bodies. • There's gotta be more doctors out there in tune with women • There's a saying: “Doctors bury their mistakes”
P154	Perimenopausal symptoms—didn't think it'd be cancer. Put mirena in to deal with bleeding (bled for 3 months), cut out polyps and fibroids in uterus. Anxiety.	Holding my breath for 6 long, short weeks. Looking back, I needed support. Mind racing—Anxiety. Loss of sex drive. In 6 weeks gap—No information; was asking nurse sister questions. Fear of unknown in that 6 weeks between referrals. “Oh my gosh should I be writing wills?”	“It's just a hysterectomy”—I took the cancer out of it. It's so much more than a hysterectomy—What will my partner feel in there, I'm not whole, am I womanly enough? Took ovaries—Gay and didn't want kids. “It was more of a mental battle. Like I know it was only stage one, nothing major” “I'm so lucky…”	Phone call “good news” no more cancer found—Then I broke down. Had internalized it all for too long. Follow‐ups: fear of recurrence.	Lesbian context—Sex and cancer book. Internal examinations—Not used to things up there. Intimacy concerns: “Didn't want to be touched because I didn't know what was happening inside me”. Am I still womanly enough because I don't have the bits (loss of parts of self? Girly bits gone). “I internalized it for too long and not sought help”. Should have contacted someone for information.	• Emotional impact—“Scared to call cancer council”—Am I jumping the gun? Am I overreacting? Only stage 1 (comment from dad). Needed the support normalized from medical team. • “It was sorta all medical… there was no sorta ‘if you need to talk to anyone, or if you need any more information’” • They say knowledge is power, but sometimes my anxiety it's worse for me to know. • Fear of recurrence—Hormone replacement therapy (HRT) patches and 8% higher chance of breast cancer—Stuff that didn't cross my mind, it wasn't spoken about or anything. • Tried not to question too much further ahead for my anxiety kind of thing • Approaching 1 year anniversary

Assumptions became an analytically useful term for BW to hold our interest in latent patterns in the data (i.e., socially constructed ideas that were taken‐for‐granted) and hence were chosen over the usual terminology of ‘themes’. Assumptions, from a constructionist lens, are not owned by individuals but rather part of the socially constructed and circulating ‘truths’ (about ‘the way things are’). BW's decision to follow what was of interest in the data was also informed by her critical and reflexive stance in which she was avoiding a privileging of ‘proceduralism’ or ‘methodolatry’ implying there is a ‘right way’ to thematic analysis [36, 37]. A critical, reflexive and feminist approach enabled us a constructionist reading in which language is analyzed as a site for meaning‐making and power [33, 37]. BW became interested in what counts as information, who are the sources or holders of such information, and other such assumptions women were negotiating as they shared their stories. A focus on assumptions enabled us to showcase the complexity in the data, for example, how assumptions were not always met or upheld in women's accounts, and yet they were implicit in shaping women's accounts. In what follows, where possible, we illustrate the diversity within each assumption and highlight ‘exceptions to the rule’, showing how women negotiate, resist or uphold these assumptions.

## 3 Results

We found the Australian healthcare system played a key role in shaping how women received, asked for, and understood information. While a few participants (*n* = 3) described largely positive healthcare experiences and interactions with health professionals, most recounted varying degrees of difficulty, distress, or concern with accessing information from pre‐diagnosis through to treatment. Women commonly reported challenges in understanding, integrating, and remembering information; knowing when and how to ask questions; and identifying what questions mattered. The value placed on information was highly contextual, shaped by emotional, social, and practical factors. Information was not simply technical or neutral; it was always situated in relationship. In what follows, we explore *three assumptions* women negotiated as they shared their experiences of information: (1) the physical body is of central importance; (2) more information is better; and (3) medical knowledge is most credible. These assumptions were not always upheld, at times resisted, and ultimately shaped how women narrated their information experiences. Example quotes are presented below, with additional illustrations in Table 2.

**TABLE 2:** Example quotes relating to identified assumptions.

“Physical body is of central importance”	“More information is better”	“Medical knowledge is most credible”
“I sort of went through menopause […] And then I sort of had a good 18 months and then, and then I started getting, um, lots of, um, just erratic, like I'd have 3 months in spotting and then spotting and a period for like 2 days. And it was just for the next, I suppose, 2 years it was completely all over the place. And I kept going back to the doctor, uh, who I'd seen for probably the last, you know, 25 years, and male doctor, and he just said, ‘well, that's just normal.’ And I said, ‘well, what can I do about it?’ And he said, ‘nothing.’ […] I got to a point I was getting so frustrated I couldn't see a female doctor, could only see him, a‐ a‐ a‐ a male doctor. So, a few times I went to him and was saying the same thing and it was just all over the place and he just said, ‘well, that's just life, isn't it? for a female at your age?’ you know, I was getting that type of comment all the time.” (P145)	“When we went down to the doctor's room, we went in and there was another doctor, like full dressed up. And you could tell he had just been in doing something serious ‘cause he still had, like, the glasses imprint on his face and he just looked exhausted. And they sort of just started asking questions about my period, what my history was like with that, and if I had ever had any concerns about it. Um, and that's when they finally told me. I think one of the best things the gynecologist did was, he drew me a picture. Because I have no idea about body parts and what they look like and who does what and anything like that. And I'm so oblivious to that sort of thing. But for him to, he sat there, he drew me the picture. He labeled everything. Like still to this day, year, nearly a year and a half later, I still have that picture. It just sits in my wallet. So that way I could, I actually had something to look back on to recount what he said, what it was, where it was. I think, I think that always for me was super important, that he took the time to really explain it to me.” (P157)	“So with me, as soon as … I don't know. It's nearly like a sixth sense or something. I‐ I‐ I really don't understand because, um, all my cancers have started with me just not in pain or anything, just, um, I go to the doctors and just go, ‘There's something wrong. I can't wo‐ you know I can put my finger on it, but there's something.’ And then they just run tests, and bingo, it's, um, cancer. […] There's something, there's that sixth sense. And I think every woman's got it. That something's not quite right. You know?” (P158)
“I left work because I‐ I was too emotional. Um, as you know, you're‐ you're fine, and then all of a sudden someone … as innocent as someone comes up, ‘so how are you?’ And you just break down. And then you're like, oh, I wish people wouldn't ask that question when they see you, you know? Um, so I had to give it up because looking after the elderly, they felt … They feel the pain, they wanna help. And um, yeah, I couldn't put ‘em through that, so.” (P158)	“I was considered a cat[egory] one, but there was a little bit of, uh, you know, not exactly the right information for them to start with. So the biggest issue for me all the way along the line was, um, waiting, ‘cause I'm a very proactive person’. And the waiting, for me, ‘cause of my mental health, was pretty full‐on’. So I started with information. I looked at, um, cancer council information, and p‐ pretty much tried to stay away from anything else, ‘cause I just didn't wanna go down any real rabbit holes until I sort of knew what was going on.’” (P176)	“Faith means that you trust god for what's to come, you know it's not going to stop you from anything bad. But you know someone's gonna be with you. And I've had one op where, uh, let's say I had a bloop and I thought of some… ended up having a conversation up there. And it was, ‘if you'd taken me on the first one, then I would've understood but this time I'm really angry.’ (laughs). And it just sort of came too when I said, ‘I choose life.’ And then I was back. So, whether you regard that as hallucination or what I don't know but, um, yeah it was just, um, yeah, I guess an experience, um, it didn't promise me lots of life, didn't promise me an easy life. But it is my life and it's a life that I live with god.” (P120)
“The brain fog is unbelievable. I can't remember half of it. It's just my brain is overwhelmed, absolutely overwhelmed. Um, I was already suffering from anxiety, um, and PTS, mild PTSD beforehand. It just… that got worse. Um, before the cancer, like, that was already there and, and everything. That was diagnosed 2 years before the cancer, so I'm still trying to get them under control. […] I had a bit of that [overwhelm], a bit before I was diagnosed [with cancer]. That was part of the anxiety. It just got to a point where I couldn't mentally think straight. It's gotten worse, um, since I had the cancer. But I, I just think it's all from, you know… It is very overwhelming. My, my brain is just going. I need to stop.” (P153)	“When I went for the hysteroscopy I was nervous but I‐ I was nervous more about the, um, thought of being‐ I don't like going under. I don't like being un‐ under anesthetic‐ anes‐ general anes‐ anesthetic. Uh, I worry about not waking up. So, anyway, when I woke up from that procedure the doctor approached me. Um, I just literally woken up. Like I'd only been awake for probably half a minute. 30 s. The nurse just said how are you feeling? I said, um, yeah, um, okay, and I‐ I didn't even think anything. I just, you know, I was just waking up. I thought I'm alive, that's great. I've woken up from surgery and then he comes over to me and he just says, the‐ the doctor or the surgeon, it's cancer, it's bad. Um, you're going to need, uh, radiotherapy and‐ and most likely chemotherapy as well. That's what he said to me. […] I just knew my gut instinct told me if I was to do what the doctor's said I would die. And there was no‐ it was just an absolute certainty that I would die. And this was before I did the research into it and really found out the‐ the har‐ the‐ like how conventional treatment really is‐ it's about money. They get a lot of money from, uh, um… they get a lot of money. The pharmaceutical industry and the doctors too, they get big kickbacks when they recommend chemo, in particular. I'm not sure about radiation. Possibly, but definitely chemo. (P148)	“Yeah, I am curious about the MRI. I'm a facts girl and I like to get all my ducks in a row. Um, I even changed my will just after I was diagnosed again. Um, you know, so you do think about things like that as well.” (P166)
“The mental stuff ‘cause I didn't realize how much you know, that was affecting me at the time’ ‘cause, um, when the gynecologist inserted the mirena’, she said, ‘look, if this doesn't work, in 6 months we're gonna do a hysterectomy.’ I thought, ‘okay, no worries.’ but then sh‐ um, you know, when I went back and she said, ‘well, you've actually got cancer so that hysterectomy has to happen now,’ I went, ‘oh, yeah, it's just a hysterectomy,’ you know? It's what I was having 6 months down the track… is happening now. And you know, it sorta took the cancer out of it. And then I don't know if it's just the, the hormones missing now I'm in menopause or what, but like, shit, it was so much more than a hysterectomy.” (P154)	“You know, they say, ‘knowledge is power,’ but sometimes my anxiety, it's, it's worse for me to know.” (P154)	“I'm just so glad I did my research because I did… after my diagnosis, I kind of had it in my head I wasn't going to do the chemo and radiation but the surgery was something I wasn't sure about. Um, but then I did more‐ more research, um, found some stuff online then I found some books and I actually, um, I just decided within 1 week. It only took 1 week's research that I wasn't going to do it. I was still on the fence about the surgery, um, but then as time went on I decided against that as well.” (P148)

### 3.1 The Physical Body is of Central Importance

This assumption encapsulates the notion that physical symptoms and the physical body are of priority within a healthcare system that diagnoses and treats such symptoms. While women did not state their experience in such explicit terms, as is the nature of a cultural assumption, instead they noted tracking physical symptoms as a way of recognizing something was amiss and seeking help from their doctor. As women shared their experiences of pre‐diagnosis and diagnosis, the emphasis was on their symptoms of bleeding, pain or discomfort. For those that had health professionals who took their physical symptoms seriously, the diagnosis process was somewhat straight forward. Women experienced difficulty in the early stages when health professionals were described as not taking symptoms seriously or with concern. In these latter instances, the lack of concern was more often reported to come from male doctors who were said, for example, to dismiss bleeding as ‘normal’, associated with age or being female (see P145 in Table 2).

Another way this assumption was evident was in women's frequent descriptions of their emotional experiences being overlooked. This included reports of health professionals' lack of attention to the psychological and social impacts of diagnosis and treatment. For example, women spoke of needing someone to talk to or debrief with, someone to share the mental and emotional load of cancer (see P153 and P158). For many women this role was filled by partners or husbands, or close friends or family who joined them at appointments. Based on women's experiences, the ‘side‐effects’ of cancer frequently went beyond the physical to include a range of psycho‐social impacts, such as distress, anxiety, post‐traumatic stress disorder, panic attacks, and depression. Some women described leaving their job or having to reduce their engagement with social or personal activities because of treatment. However, no women spoke of these potential impacts being acknowledged as part of information they were provided during their treatment. Only a few women reported that they felt *emotionally* supported by their medical team (e.g., P157 and P176). For example, several women described having to use dilators following radiation treatment as ‘embarrassing’, finding that the education and hand‐over was not held sensitively. These women described receiving a dilator with little warning in advance, one noting that she would not have had radiation if she knew she had to use a dilator. The dilator example offered insight into the potential sensitivity required for informing and supporting women post‐treatment.

As an exception to the data, and another illustration of the ubiquity of this assumption, one woman (P148) was diagnosed after a hysteroscopy and described declining any further medical treatment for her cancer. She approached her cancer from, what she framed as, a *holistic* lens: considering her whole life and way of living as contributing to the cancer. Thus, to her, treatment involved more than the physical body and required addressing root causes to disease in the body (her childhood trauma). Through her cancer journey, she described a backlash from health professionals and felt unsupported by the medical establishment. She described how since changing her life (including leaving her stressful job, changing to an organic vegan diet, having colonics, and taking care of herself, seeking psychological care) her physical symptoms (i.e., bleeding and pain) had stopped. Hence, even though she resisted treatment in its medical form, she ultimately still drew on this assumption (i.e., her lack of physical symptoms) to point to the value of her holistic approach. This participant's story shows how an assumption can be both resisted and upheld.

### 3.2 More Information is Better

The second assumption deals with the underlying idea that more information is better than less information. Conducting research on gynecological cancer information experiences, it is possible that women were responding not only to their own local contexts but also our inquiry as researchers. Hence, we found women generally wanted information about what to expect, practical information about how to manage their care at home (e.g., about dilators), and how the frequency of follow‐up/contact will change and why. They also generally wanted this information to be explained earlier so that they had time to prepare. It seemed there was no dispute that quality information that women can understand and engage with (e.g., when telling family or friends about their cancer) was important. However, informed by a feminist and critical approach, we sought to probe beyond the idea of ‘more information is better’, and in doing so we were able to hear that when women had received information, they did not always understand it nor felt supported to integrate and remember the information. For example, some women were not aware of or understood the meaning of their diagnosis, nor were clear on treatment side‐effects, what happens after treatment, and/or the risk of recurrence. In an attempt to hear beyond the initial ‘more information is better’, we identified two nuanced ways women negotiated this assumption: a *capacity* for information and the *patient‐provider relationship*.

Women's *capacity to understand information* provided by health professionals at appointments or online was an implicit aspect of their interviews. We noticed this from looking at how women spoke about conversations with their health professionals regarding diagnosis, including where their use of the word ‘cancer’ had the potential to de‐stabilize and, in some cases, overwhelm women. Women's language exemplified the psychological impacts of these conversations, for example their “brain turning to gel”, their “head is in a tumble dryer”, or their “mind going 100 miles an hour”. In addition to this, many women reported experiencing brain fog after treatment, such that a few women opened their notebooks during the research interviews to remember the details of their experiences. In these cases, women shared approaches they used to manage the potential for forgetting or overwhelm, such as taking notes during appointments or bringing someone along to appointments. In short, we found that information around cancer was not neutral for women. Information was framed in both positive (“Booklet helped me explain it to my family” P157) and negative terms (“I am mentally exhausted” P153). Hence, information itself was contextual as was women's capacity to receive and engage with the information and how it was delivered/accessed, thereby adding complexity to the idea that ‘more is better’.

Many women spoke about the *importance of their relationship* with their General Practitioner—or in some cases, their specialists. This included the value of having their test results ‘translated’ or explained into the bigger picture of their health history. This was particularly important in the early stages leading up to diagnosis or treatment, where this relationship was crucial in delivering time sensitive information. However, several women did not have a trusted professional to do this with or lacked the continuity of care in terms of a health professional who they saw for appointments. Inherent in these references in the data was the need for a relational component to women's treatment. That is, an appreciation that information is conveyed and received within a relationship that holds the needs, context, priorities and history of the patient. For women who had a strong relationship with a health professional, their experiences were often framed in positive terms. This nuance suggested that information is relational and a strong relationship may enable ‘more’ to indeed be ‘better’.

### 3.3 Medical Knowledge is Most Credible

The final assumption is concerned with the implicit notion that certain sources of information or knowledge systems were deemed more credible. Consequently, women did not give all information given equal legitimacy. Specifically, we found that across the interviews, women positioned medical knowledge as the most trusted source of cancer information, often disseminated through online resources, pamphlets, or directly from health professionals. These sources allowed women to translate their diagnosis and treatment for themselves and their families. While women rarely questioned this trust in medical knowledge, they did describe supplementing such information with additional sources of knowledge referencing holistic practitioners, spiritual frameworks, and embodied or intuitive knowing, many of which supported women's emotional, existential, or relational needs. Within this assumption, it was at times difficult to disentangle information from knowledge, as what counted as credible information was shaped not just by the content itself, but by who shared it and the systems of expertise it came from.

One of the common ways in which we identified this assumption was in relation to who was granted ‘expertise’ in women's accounts. Overwhelmingly, we found that women allocated trust to medical sources of knowledge and medical professionals who communicated the information. We found women spoke unproblematically and unquestionably about information from medical sources: its status as truthful and trustworthy was rarely challenged, with one exception. In this case, the participant distrusted medical advice and research and put her trust in lived experiences of others and holistic health professionals (P148). While a few women questioned or acknowledged the problems of the medical system, other than P148 none challenged medical knowledge in its trustworthiness. In the majority of instances when the problems of the system were acknowledged, with language such as “health system is like a cattle yard” (P149), “doctors bury their mistakes” (P158) or “you're just a number” (P138), the common thread seemed to be the extent to which women noted their lack of visibility as a patient with unique needs.

Although very few women questioned the legitimacy of medical knowledge, women often went beyond medical information to support their treatment journey. Several women sought alternative or holistic information and in so doing prioritized their health and wellbeing beyond the physical treatment and side‐effects (see first assumption). These examples showed the breadth and depth of information women were seeking over the course of their diagnosis and treatment. This included psychologists, counselors, therapists, and naturopaths. Some women described a higher power or presence in their life, with whom they could trust for insight and perspective in the face of the existential threat of cancer (P120, P148 and P140). While evident in a minority of the data, these examples circle back to the notion of trusted sources of knowledge, such that these participants allocated trust and legitimacy in a higher power.

Finally, we also noticed women's references to their own embodied or intuitive knowledge as a source of credible information. This was described as “gut instinct” (P148), or “sixth sense” (P158) or, in more gendered terms, “women have gotta listen to themselves and their bodies” (P158). Such references were evident in only a few interviews. This notion of trusting that women know their bodies was also evident in those few interviews where women spoke of being dismissed by male doctors. This inner ‘source’ of knowledge or information was less common in the data and perhaps illustrates the positioning of medical professionals as trusted sources.

## 4 Discussion

This study is based on 24 interviews with women who were diagnosed with endometrial cancer. This critical, reflexive thematic analysis focused on women's information experiences with an interest in women's language as socially constructed. Hence, we explored three assumptions women were negotiating as they described their experiences of information: (1) that the physical body is of central importance; (2) that more information is better; and (3) that medical information is most credible. We showed how assumptions were not always met or upheld by women, and at times resisted.

The first assumption, that the physical body is of central importance, was evident in women's descriptions of the physical symptoms that helped them secure a diagnosis. This assumption was also evident in women's accounts of the absence or lack of acknowledgment of their emotional experiences. This assumption aligns with prior work showing that women's cancer diagnosis and treatment involved a range of complex and often unexpected psycho‐social impacts for women and their lives [8, 38, 39]. The second assumption, that more information is better, has also been problematised in the literature, with studies noting that cancer patients walk a fine line between information seeking and information overwhelm [17]. Consistent with this, women in our study highlighted the importance of having a trusted patient–provider relationship in which information could be interpreted and held, rather than being left to manage it alone or to be overwhelmed by it. The final assumption concerned what counts as ‘trusted’ sources of information. Echoing prior research [8, 40], women overwhelmingly privileged medical knowledge, while also weaving in alternative or holistic sources of support. Our findings add to this body of work by emphasizing how women dynamically combined medical expertise with subjective, experiential, and relational forms of knowing. Rather than questioning the value of medical authority, these results point to the importance of recognizing how patients draw together multiple knowledge systems to make sense of illness, especially when relational, emotional, or existential needs are at play [9].

This study offers a nuanced account of women's experiences of information across their endometrial cancer diagnosis and treatment, with contemporary relevance given the recency of participants' diagnoses. Journey mapping offered a rich way to capture how women told stories of information over time, also providing a way to support early analysis (Table 1). A feminist methodology supported a consideration of power throughout the study, from privileging patient‐led storytelling [29] to an interest in the implicit and taken‐for‐granted meanings within the data [33, 37]. An unintended but beneficial impact of the interviews was that women reflected on how the interview itself helped them formulate questions for their doctors, hear their own voices, and recognize their strength, underscoring the therapeutic potential of research encounters.

We received feedback from participating women about the survey completed prior to the interview. A key point was the need to better acknowledge women's emotional realities. Women suggested the survey to feel less ‘clinical’, both in language and visual presentation, so it did not resemble a hospital survey.^3^ They also wanted the emotional impacts of their experience, including sexual wellbeing, to be more fully reflected in the questions. We share this feedback to encourage future researchers to consider the psycho‐social framing and emotional impact of their research materials.

### 4.1 Study Limitations

While the sample primarily included women diagnosed at an early stage, reflective of the broader clinical population, findings may not reflect psycho‐social impacts faced by women with advanced, metastatic, or recurrent disease. In addition, while having women complete a survey prior to interview offered insight into their informational needs, it may also have shaped how participants reflected on their experiences, and also who chose to be interviewed. Although not intended to be generalizable, the findings may have relevance for other gynecological cancer contexts, while recognizing that distinct cancer types may involve unique informational and psychosocial challenges.

### 4.2 Clinical Implications

Situating these findings within a *patient navigation model* [10] highlights the need for clinicians to critically reflect on how assumptions about ‘information navigation’ (what is relevant, how it should be delivered, and who is considered a credible source) are embedded in cancer care. Health professionals play a central role in facilitating access to contextually sensitive information that supports not only physical health, but also psycho‐social wellbeing. This includes recognizing patients' diverse information preferences, emotional readiness, and alternative sources of knowledge. These findings align with the priorities and perspectives outlined in the Australian Cancer Plan foregrounding person‐centered care [41], as well as international guidelines informed by similar principles [26, 27, 28]. Further research is needed to explore health professionals' perspectives and experiences of information‐sharing, particularly regarding perceptions of the psycho‐social impacts and patient experiences. There is also scope to evaluate the implementation and effectiveness of patient navigation strategies that assist with information engagement, such as text‐based symptom‐monitoring programs and communication aids [42]. Notably, several participants reflected that taking part in the interview helped them clarify questions they wanted to ask their care team, suggesting that structured reflection and guided prompts could have value as part of routine care. Future research should examine the feasibility and acceptability of such tools, with attention to enhancing relational communication and shared understanding in gynecological cancer care.

## 5 Conclusion

Women's accounts of information experiences across their endometrial cancer diagnosis and treatment were shaped by complex emotional, relational, and contextual factors. This study highlights the need to critically reflect on the assumptions that underpin cancer care, and the importance of delivering information in ways that are both clear and contextually responsive. How, when, by whom, and in what form information is shared can significantly shape women's understanding, their capacity to engage with information, and the sources they come to trust. Meaningful information‐sharing requires not only accurate content, but also continuity of care and opportunities for dialog and relationship. These findings may inform future efforts to strengthen communication and support across gynecological cancer care pathways.

## Author Contributions

B.W., A.D., B.B., J.C., and T.D. contributed to conception and design of the work. B.W., and T.D. contributed to data collection. B.W. conducted data analysis and interpretation and led the drafting of the work (with support from T.D., and A.D.). All authors contributed to critically revising the manuscript for publication. All authors agreed to be accountable for all aspects of the work in ensuring that questions related to the accuracy or integrity of any part of the work are appropriately investigated and resolved. All authors read and approved of the final manuscript.

## Funding

This project was supported by the Australia New Zealand Gynecological Oncology Group (ANZGOG) and funded by AstraZeneca. JC was funded by a National Health and Medical Research Council (NHMRC) Research Fellowship (#1058244).

## Ethics Statement

Approval was obtained from the Human Research Ethics Committee of The University of Queensland (HE000046). The procedures used in this study adhere to the tenets of the Declaration of Helsinki.

## Consent

Informed consent was obtained from all individual participants included in the study.

## Conflicts of Interest

T.D., and A.D. were awarded by the Australia New Zealand Gynecological Oncology Group (ANZGOG) funding from AstraZeneca. The funder had no role in the design of the study; in the collection, analyses, or interpretation of data; in the writing of the manuscript; or in the decision to publish the results. There are no other conflicts of interest.

## Supporting information


Supporting Information S1

## Data Availability

The data that support the findings of this study are available on request from the corresponding author. The data are not publicly available due to privacy or ethical restrictions.

## Endnotes

^1^
It was examples such as this (the “right” participant) that oriented to the implicit power dynamics that were part of the research project and that the first author took to documenting in her field notes. These observations are part of the feminist lens through which BW undertook data collection and analysis, and the eventual focus on *assumptions*—a way to study the power reflected in language use, in the context of research interviews about gynecological cancer information.

^2^
We have chosen to use double quotation marks to quote direct speech and single quotation marks for our own analytical purposes.

^3^
That said, one participant (P140) described participating in a clinical trial and found that the survey she filled out as part of the trial was a very useful form of information.
