# Supplementary material for: Exploring Women's Experiences of Information Across Their Endometrial Cancer Diagnosis and Treatment: A Qualitative Analysis
Source: Psychooncology. 2026 Apr 1;35(4):e70444. doi: 10.1002/pon.70444 (PMC13041597; doi:10.1002/pon.70444)

**Supplementary Material S1: Interview Guide and Journey Map**

**Interview structure:**

1. General introduction

2. Interview questions to begin the conversation

3. Patient journey mapping

4. Opportunity for ‘anything else’ question

5. Referral to support services

6. Thank women

**1. General introduction:**

Thank you for agreeing to participate in this interview today. The aim of today’s interview is to understand your experiences leading up to and since being diagnosed with endometrial cancer. We are interested in how you were feeling, what you were thinking, and where you sought information from during your cancer journey. In particular, we would like to know what information you needed at different phases of your cancer journey and if you were able to access this information.

The interview will be more like a conversation, I’ll ask you about your diagnosis with endometrial cancer. By sharing your experiences with us, you will help ensure that the right information is provided at the right time to gynaecological cancer patients throughout the cancer journey. I expect the interview will take around 60 minutes.

Before we begin, I’d like to confirm some key information:

- Your participation is voluntary;
- You are free to withdraw from the interview at any time without penalty;
- You are free to decline to answer any questions you don’t feel comfortable with, we can also take breaks or reschedule the interview for another time if you prefer;
- Your interview is completely confidential and all information you provide will be de-identified;
- While you are unlikely to experience any direct benefit from participating in this interview today, your participation may help other women who have gynaecological cancer in the future;
- Finally, your interview will be audio-recorded. The recording will be stored securely and only accessible to approved members of the research team.

Before continuing, can I please confirm that you agree to participate in this interview today?

**2. Interview questions to begin the conversation:**

The questionnaire you completed asked about your information needs during your cancer journey. Tell me about your own cancer journey – provide a brief overview from early on until now, and we will come back for details.

**3. Patient journey mapping:**

We will now go over your history with endometrial cancer and gradually advance through the different phases we just identified. In particular, I’m interested in hearing about your actions, mindset, interactions with health and other services, and emotions that marked each step.

**4. Anything else questions:**

- Is there anything else we haven’t touched on yet that you’d like to tell be about?


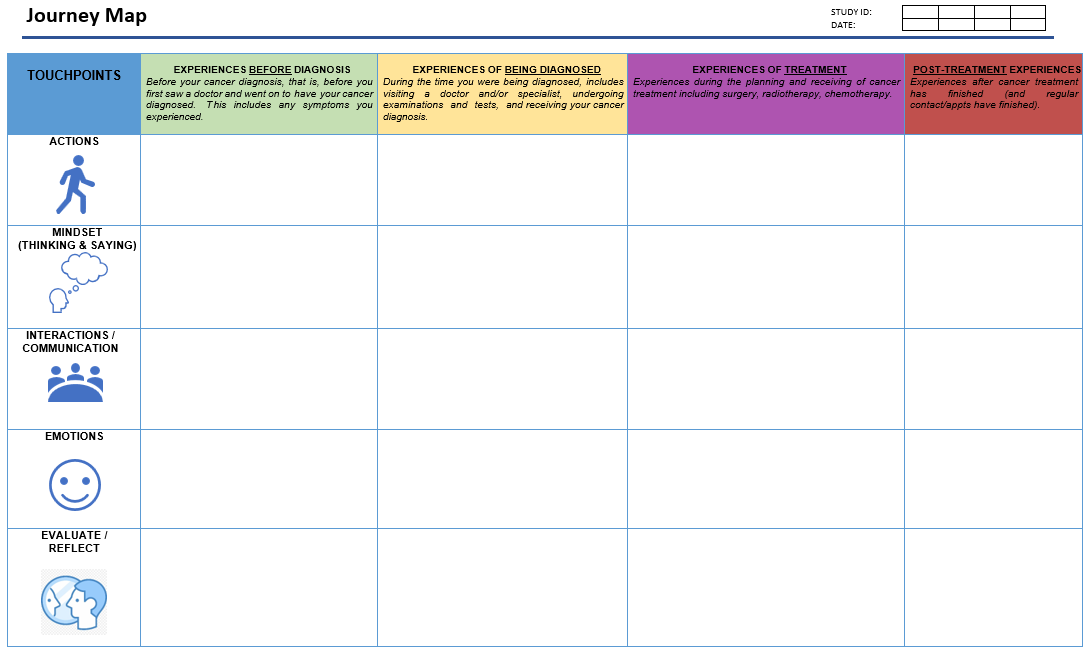

Supplement: Supplementary file 1 — Supporting Information S1 [file PON-35-e70444-s001.docx]
